# Chest pain in primary care: is the localization of pain diagnostically helpful in the critical evaluation of patients? - A cross sectional study

**DOI:** 10.1186/1471-2296-14-154

**Published:** 2013-10-18

**Authors:** Stefan Bösner, Katharina Bönisch, Jörg Haasenritter, Patrice Schlegel, Eyke Hüllermeier, Norbert Donner-Banzhoff

**Affiliations:** 1 Department of General Practice/Family Medicine, Philipps University Marburg, Marburg, Germany; 2 Department of Mathematics and Computer Science, Knowledge Engineering & Bioinformatics, Philipps University Marburg, Marburg, Germany

**Keywords:** Chest pain, Pain localization, Coronary heart disease

## Abstract

**Background:**

Chest pain is a common complaint and reason for consultation in primary care. Traditional textbooks still assign pain localization a certain discriminative role in the differential diagnosis of chest pain. The aim of our study was to synthesize pain drawings from a large sample of chest pain patients and to examine whether pain localizations differ for different underlying etiologies.

**Methods:**

We conducted a cross-sectional study including 1212 consecutive patients with chest pain recruited in 74 primary care offices in Germany. Primary care providers (PCPs) marked pain localization and radiation of each patient on a pictogram. After 6 months, an independent interdisciplinary reference panel reviewed clinical data of every patient, deciding on the etiology of chest pain at the time of patient recruitment. PCP drawings were entered in a specially designed computer program to produce merged pain charts for different etiologies. Dissimilarities between individual pain localizations and differences on the level of diagnostic groups were analyzed using the Hausdorff distance and the C-index.

**Results:**

Pain location in patients with coronary heart disease (CHD) did not differ from the combined group of all other patients, including patients with chest wall syndrome (CWS), gastro-esophageal reflux disease (GERD) or psychogenic chest pain. There was also no difference in chest pain location between male and female CHD patients.

**Conclusions:**

Pain localization is not helpful in discriminating CHD from other common chest pain etiologies.

## Background

Patients with chest pain are encountered on a regular basis in primary care. In different studies the incidence of chest pain varies according to setting, country, and inclusion criteria [1-3]. There is a wide range of different underlying diseases including coronary heart disease (CHD) [4,5].

In regard to the diagnostic work-up of chest pain patients primary care providers (PCPs) are trained to elicit, among other information, the exact pain location. Both clinical guidelines and standard textbooks recommend a detailed clinical history including pain location and radiation [6,7]. Several diagnostic studies and meta-analyses have examined the diagnostic value of pain location mainly in regard to CHD and acute coronary syndrome (ACS) [8-12]. In these studies, pain locations were normally marked on a pictogram either by patients or the attending physician. Marked areas were consequently aggregated (e.g. 'upper left pain’) for analysis, resulting in a loss of detailed data [9,10,13]. Most of these investigations were performed in secondary care settings and results are setting-specific and inconsistent.

Pain maps have been used frequently in other areas of research like low back pain [14,15], migraine headaches [16], or temporomandibular disorders and fibromyalgia syndrome [17]. While some of these studies still used conventional grid methods as the above quoted chest pain studies did [15], other authors applied advanced methodological techniques superimposing pain drawings [14] and transforming data into two-dimensional color coded images [17].

In our study we applied a newly developed technique to analyze pain drawings of a large cohort of unselected and consecutively recruited primary care patients with chest pain in order to find out whether pain localization is helpful to discriminate between CHD and other diseases.

## Methods

The primary aim of our original cross-sectional diagnostic study was to investigate the diagnostic accuracy of signs and symptoms for chest pain patients with CHD [18]. A detailed account of our study design can be found there. In this article we report results of a sub-analysis with regard to the diagnostic value of pain location in patients with chest pain.

### Participating PCPs and patients

Out of 209 contacted PCPs, 74 (35.4%) agreed to participate in the study. PCPs consecutively recruited every patient above 35 years with pain localized in the area between the clavicles and the lower costal margins, and the anterior to the posterior axillary lines. Patients were eligible irrespective of the acute or chronic nature of their complaints, including known conditions like CHD, and were also recruited during home visits and emergency calls. Patients were excluded if their chest pain had subsided for more than one month, had already been investigated, or in case of a follow-up visit for previously diagnosed chest pain. The study protocol was consistent with the Declaration of Helsinki and all participants gave their informed consent.

### Data collection and analysis

#### Baseline data

**Figure 1:**
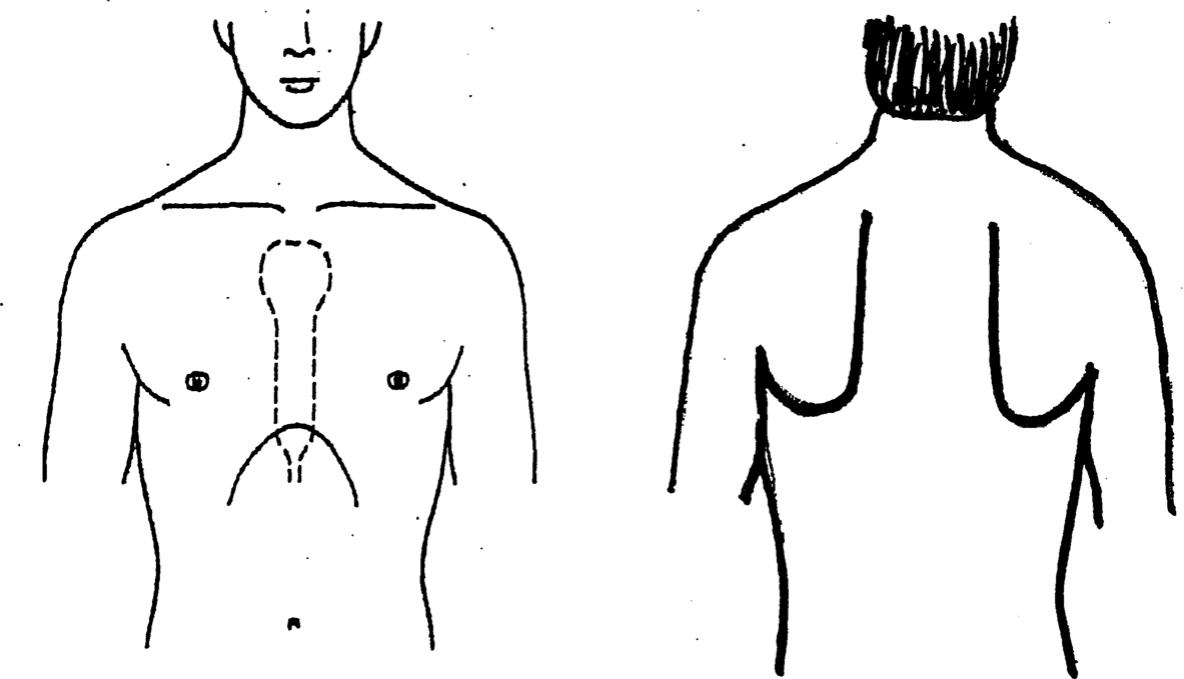
Front and rear view pictogram for data collection.

PCPs took a standardized history and performed a physical examination. On a case report form (CRF), PCPs entered the exact location and radiation of the patient’s chest pain on a pictogram (see Figure 1). We instructed PCPs to shade all areas that were painful. Other items of the CRF covered information on demographic and pain characteristics, accompanying symptoms, and CHD risk factors. PCPs also recorded their preliminary diagnoses, investigations they had ordered, and management related to the patient’s chest pains.

#### Follow-up data

Study assistants contacted all patients by phone both six weeks and six months after the initial consultation and asked about the course of the patient’s chest pain and treatment thereof. Participating PCPs requested discharge letters from specialists and hospitals.

#### Precautions against selection bias

We emphasized to all PCPs the importance of recruiting every patient with chest pain and visited PCP practices at four week intervals to check compliance with study procedures. In addition, we performed random audits to identify cases of chest pain not included in the study.

#### Diagnosis and reference standard

A reference panel including 1 cardiologist, 1 PCP, and 1 research associate from our department reviewed baseline and follow-up data of each patient. They discussed and decided on the most likely etiology of the individual patient’s chest pain at the time of the index test (delayed type reference standard). The PCP’s initial diagnosis was considered among other clinical data.

#### Digitalization of pain mapping information

To be able to perform computerized analysis on the pain region data, a computer application was created which allowed a research assistant do draw each patient’s pain regions, as well as the arrows marking radiation directions, into an exact digital replica of the pictogram on the report forms using the mouse pointer. The regions are captured in binary (black and white) images with black pixels representing a region with pain. The data generated for each patient consists of two binary images with a resolution of 900×516 pixels, one containing the pain regions, the other one the pain radiation arrows.

#### Computing process and image calculations

We superimposed all our patients’ images with the respective pain regions of a group representing the number of overlaps at each pixel position. The color of the resulting graphs reflected the degree of overlap. A large number of overlaps are represented in red colors and a small number of overlaps in blue colors. In order to be able to compare pain regions across different images, the color range was scaled to the total number of patients in the group. This means that only a region where all patients of this group overlap will be represented with the maximum possible color value (red).

#### Statistical analysis of differences between pain maps

In order to answer the original question concerning a possible distinction between diagnostic groups on the basis of their respective pain regions, individual pain localizations were compared in terms of a suitable measure of dissimilarity. Differences on the level of diagnostic groups were then analyzed on the basis of the pair-wise dissimilarity degrees thus produced.

More specifically, dissimilarities between individual pain localizations were measured in terms of the Hausdorff distance, which is a well-known and widely used measure for the distance of subsets of a metric space [19].

The comparison of two groups of patients with different diagnosis was accomplished by means of the C-index [20]. This index compares the pair-wise inter-group dissimilarities (i.e., the dissimilarity between two patients from the same group) with the pair wise intra-group dissimilarities (i.e., the dissimilarity between two patients from different groups). It ranges between 0 and 1 and assumes values close to 0 if the inter-group dissimilarities are large compared to the intra-group dissimilarities, thus indicating that the two groups can be well separated. A value of 0.5 indicates equal inter-group and intra-group dissimilarities and values close to 1 point to higher intra- than inter-group dissimilarities.

Finally, the significance of the C-index computed was determined by means of a standard permutation test. This test delivers a p-value which corresponds to the probability to obtain a smaller C-index if the assignment of the patients to the two groups is permuted in a random way.

The whole study was approved by the Ethics Committee of the Faculty of Medicine, University of Marburg. The study complies with the declaration of Helsinki.

## Results

### PCPs and patients characteristics

63.5% of practices were located in urban areas and 67% of the participating 74 PCPs were male (mean age of 49 years). PCPs included 1355 patients with chest pain, seven patients did not meet the inclusion criteria and 99 refused to participate in the study. PCPs returned valid case report forms (CRF) for 1249 patients (T0). 60 cases were lost to follow-up and 11 died, but provided enough information to be judged by the reference committee. Three early drop outs were not included. For 34 cases follow-up information was incomplete or ambiguous so that no final diagnosis could be made. Therefore, at T1 (6 months) the reference committee analyzed 1212 patients for the etiology of their chest pain; of those 179 (14.8%) patients (92 men and 87 women) were diagnosed as having CHD (see Table 1). The presented data analysis is based on the data of 1211 cases, as in one case the corresponding pictogram was not filled out.

**Table 1:** Final diagnoses in patients presenting with chest pain to their GP (n = 1212)

**Diagnosis**	**Frequency (n = 1212)**	**%**
Chest wall syndrome*	565	46.6
Coronary Heart Disease	179	14.8
Psychogenic disorders	115	9.5
Upper respiratory infections	98	8.1
Hypertension	48	4.0
Gastroesophageal reflux disease	42	3.5
Trauma	39	3.2
Benign stomach problems	26	2.1
Pleuro-pneumonia	25	2.1
COPD/Asthma	23	1.9
Other	52	4.3

*A variety of diagnostic terms like costochondritis, costosternal syndrome, sternalis syndrome, Tietze’s syndrome, rib-tip syndrome or xiphoidalgia have been used in the past to describe and differentiate musculoskeletal causes of chest pain. We decided to summarize these outcomes as chest wall syndrome.

### Pain localization: CHD vs. other diseases

**Figure 2:**
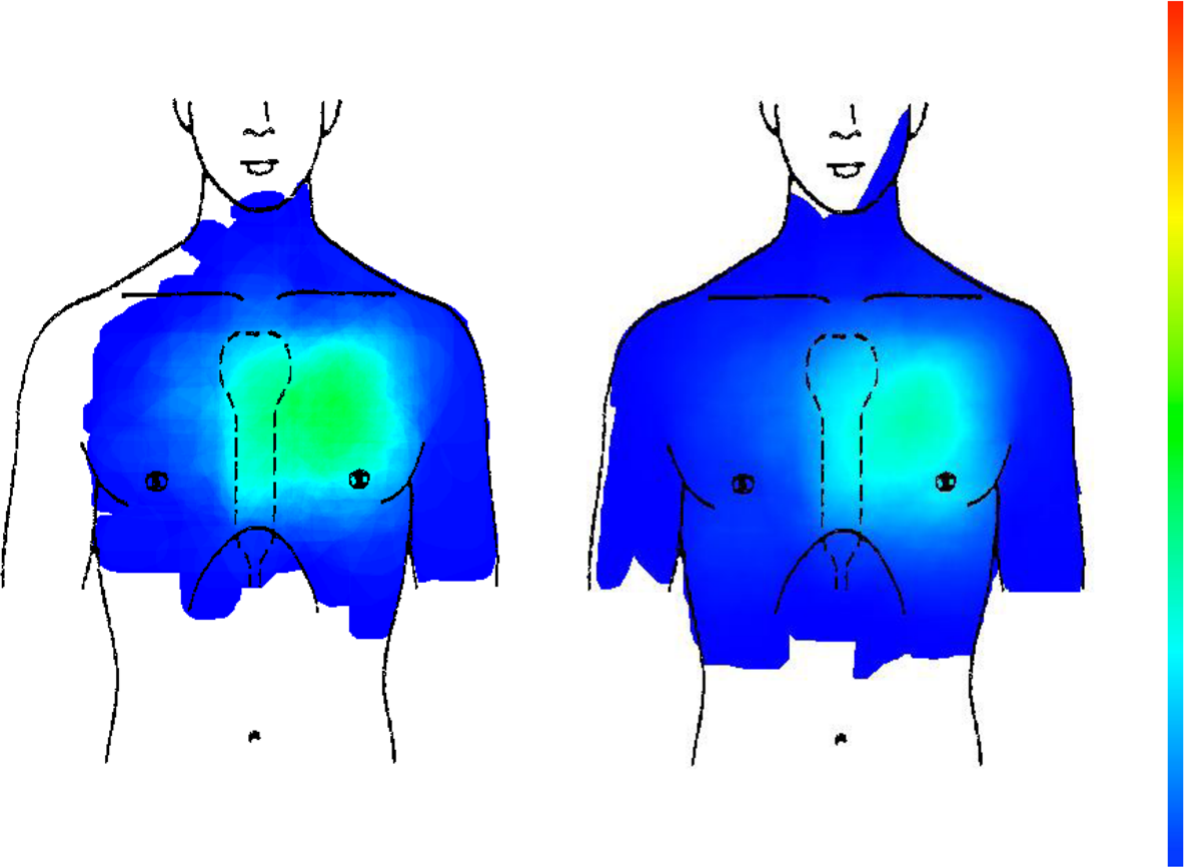
CHD (left picture, n = 179) compared with all other chest pain aetiologies (right picture, n = 1032).

In all CHD cases (179 patients) and all other etiologies (1032 patients), the pain is mainly situated in the left anterior thoracic region between the sternum and the anterior axillary line. Although CHD cases tend to concentrate more in this region, this difference is statistically not significant (see Figure 2).

**Figure 3:**
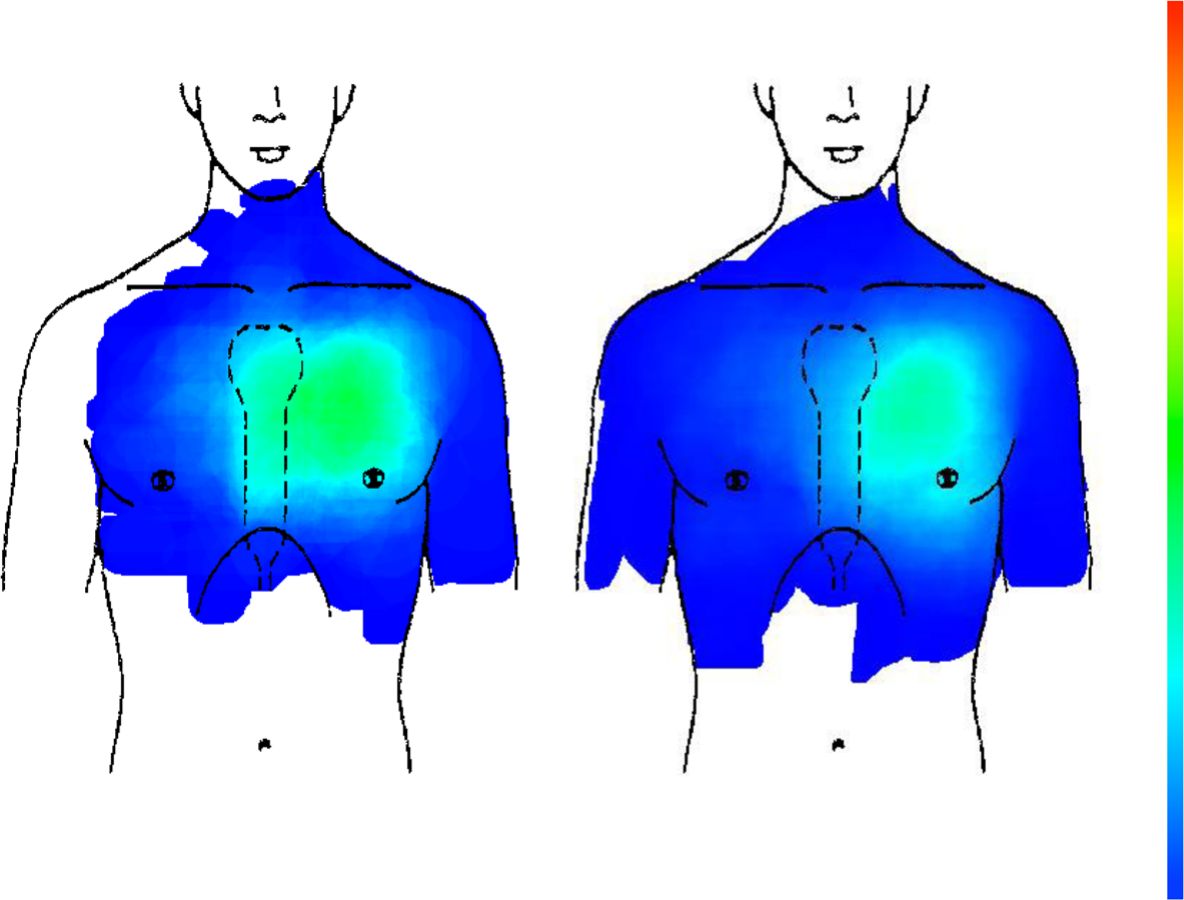
CHD (left picture, n = 179) compared with chest wall syndrome (right picture, n = 565).

Chest wall syndrome (CWS) as the single largest category (n = 565) of patients’ chest pain shows a very similar distribution as CHD. In comparison to CWS, CHD cases also tend to concentrate in the retrosternal region. Overall distribution is not statistically significant (see Figure 3 and Table 2).

**Table 2:** Comparison of different pain regions: Hausdorff-distance based clustering results

**Group comparisons**	**C-Index**	**p-value**
CHD vs. all other chest pain etiologies	0.563	0.99
CHD vs. chest wall syndrome (CWS)	0.539	0.97
CHD vs. GERD	0.504	0.27
CHD vs. psychogenic chest pain	0.498	0.30
CHD (male patients) vs. CHD (female patients)	0.496	0.140
CWS patients who assume a cardiac origin of their pain vs. CWS patients who do not assume a cardiac origin	0.450	< 0.001

**Figure 4:**
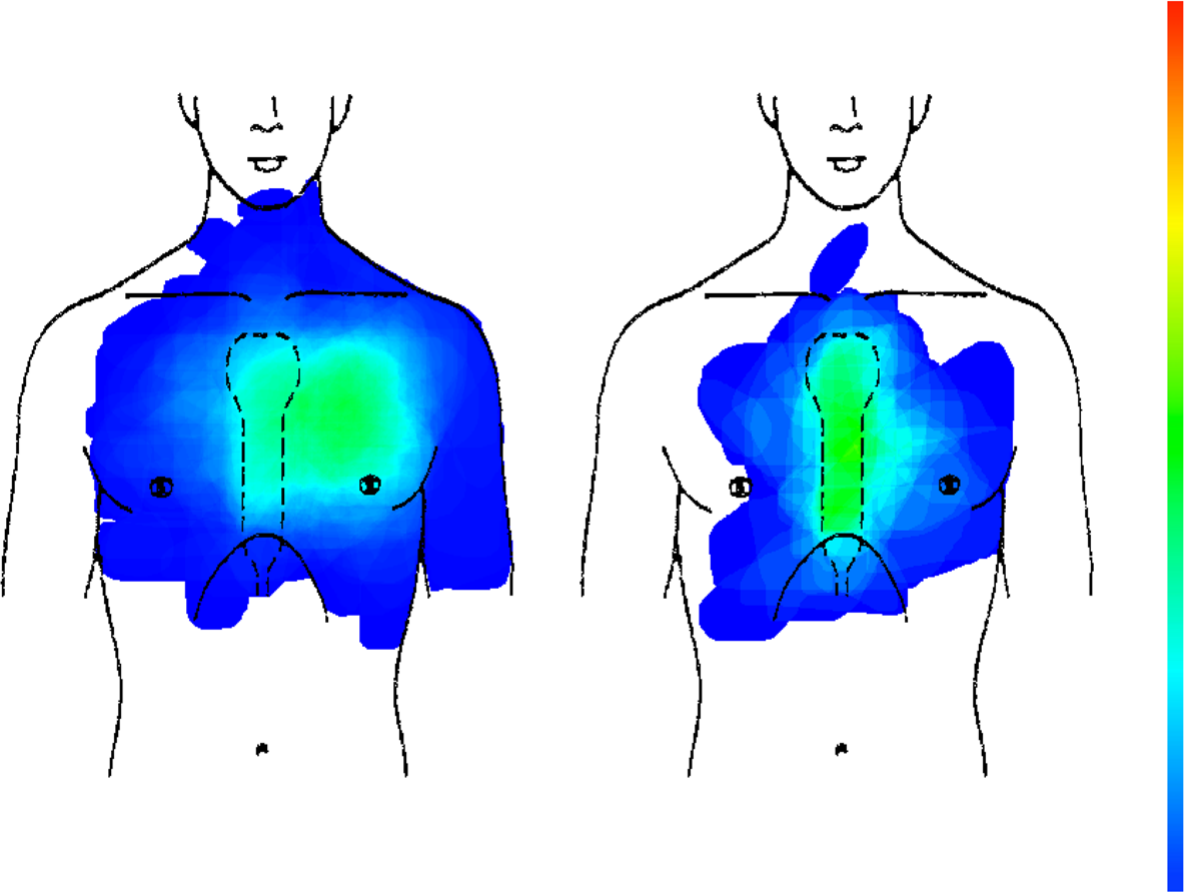
CHD (left picture, n = 179) compared with GERD (right picture, n = 42).

Compared to CHD, the pain of gastro-esophageal reflux disease (GERD) is concentrated mainly in the retrosternal region. Overall distribution is not statistically significant (see Figure 4 and Table 2).

**Figure 5:**
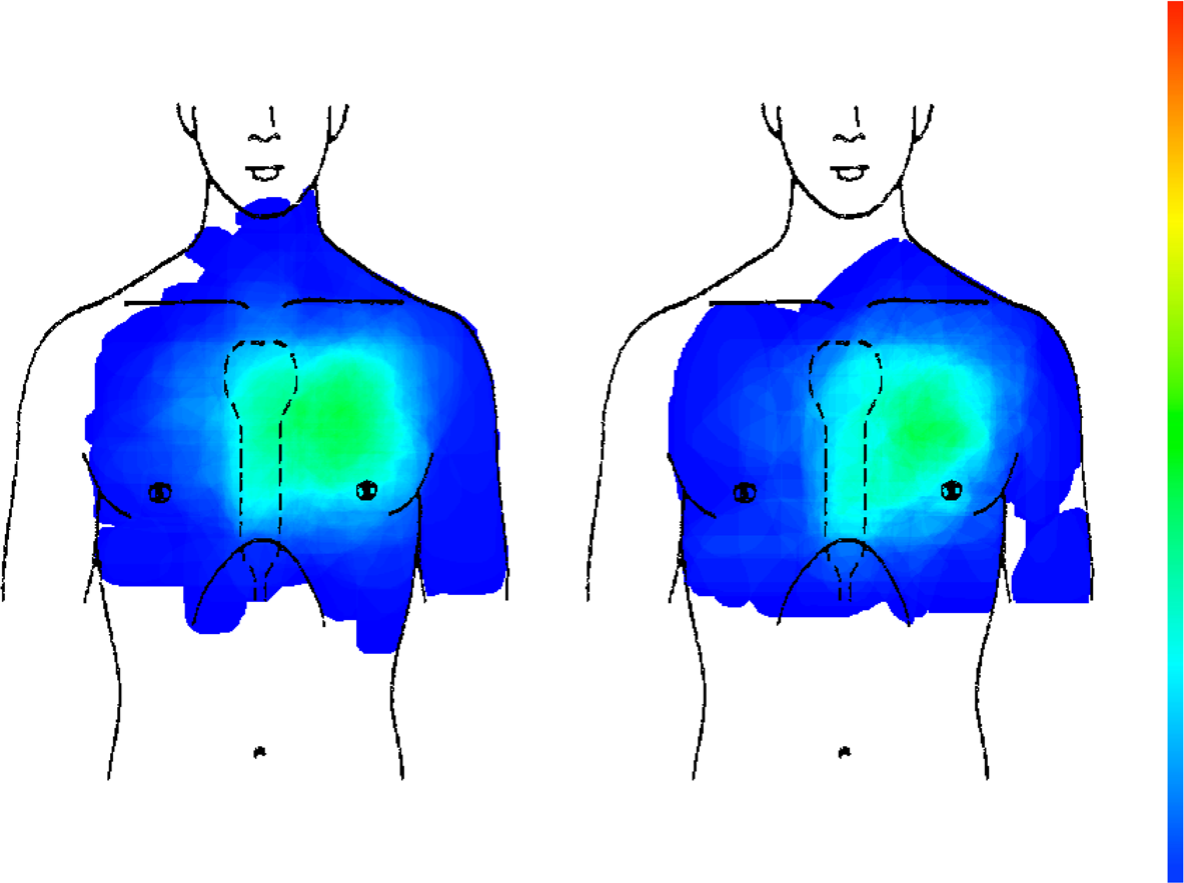
CHD (left picture, n = 179) compared with psychogenic chest pain (right picture, n = 115).

Psychogenic chest pain (n = 115) is situated mainly in the left anterior chest wall, and does not differ from CHD (see Figure 5 and Table 2).

### Pain localization: CHD by gender

**Figure 6:**
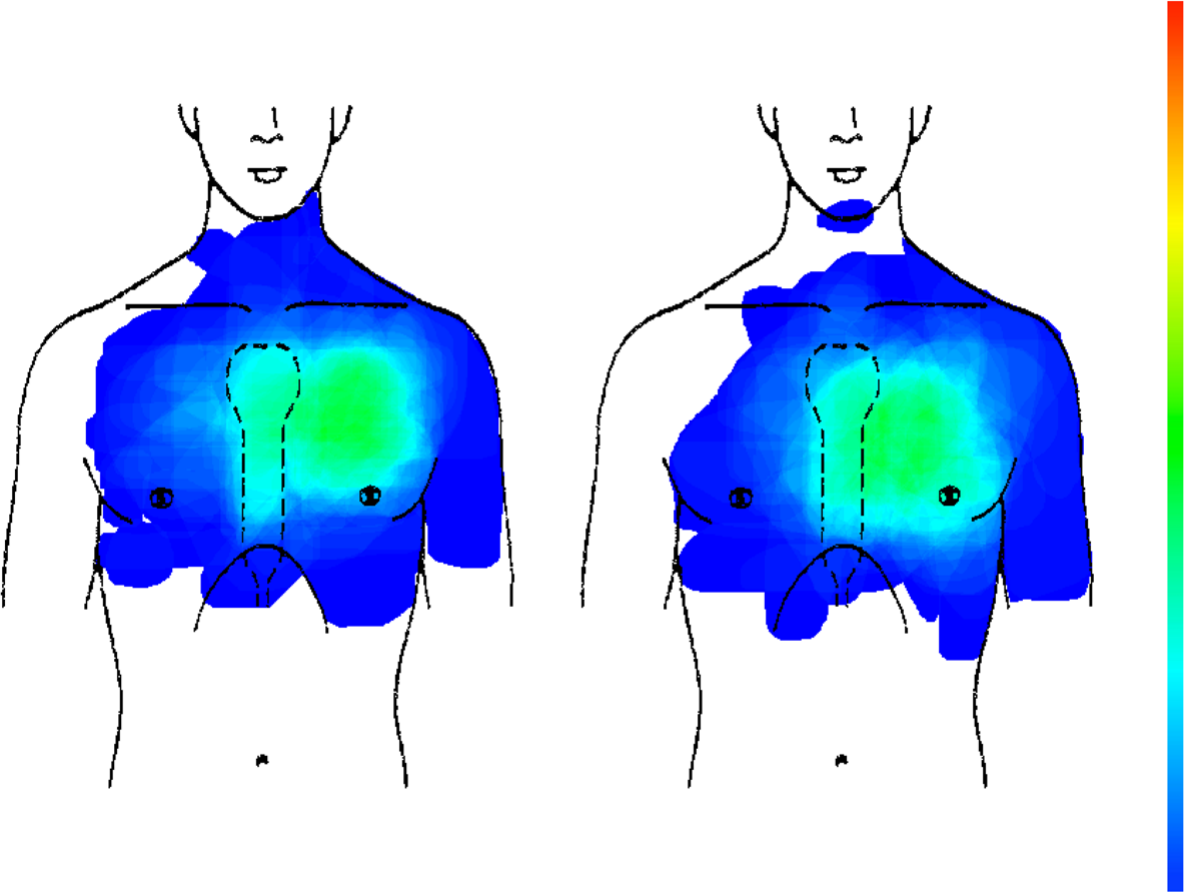
Male CHD patients (left picture, n = 92) compared with female CHD patients (right picture, n = 87).

There is no difference in chest pain location between male and female CHD patients (see Figure 6 and Table 2); for both groups pain regions are mainly situated on the left anterior chest.

### Pain localization: chest wall syndrome and patient assumptions

**Figure 7:**
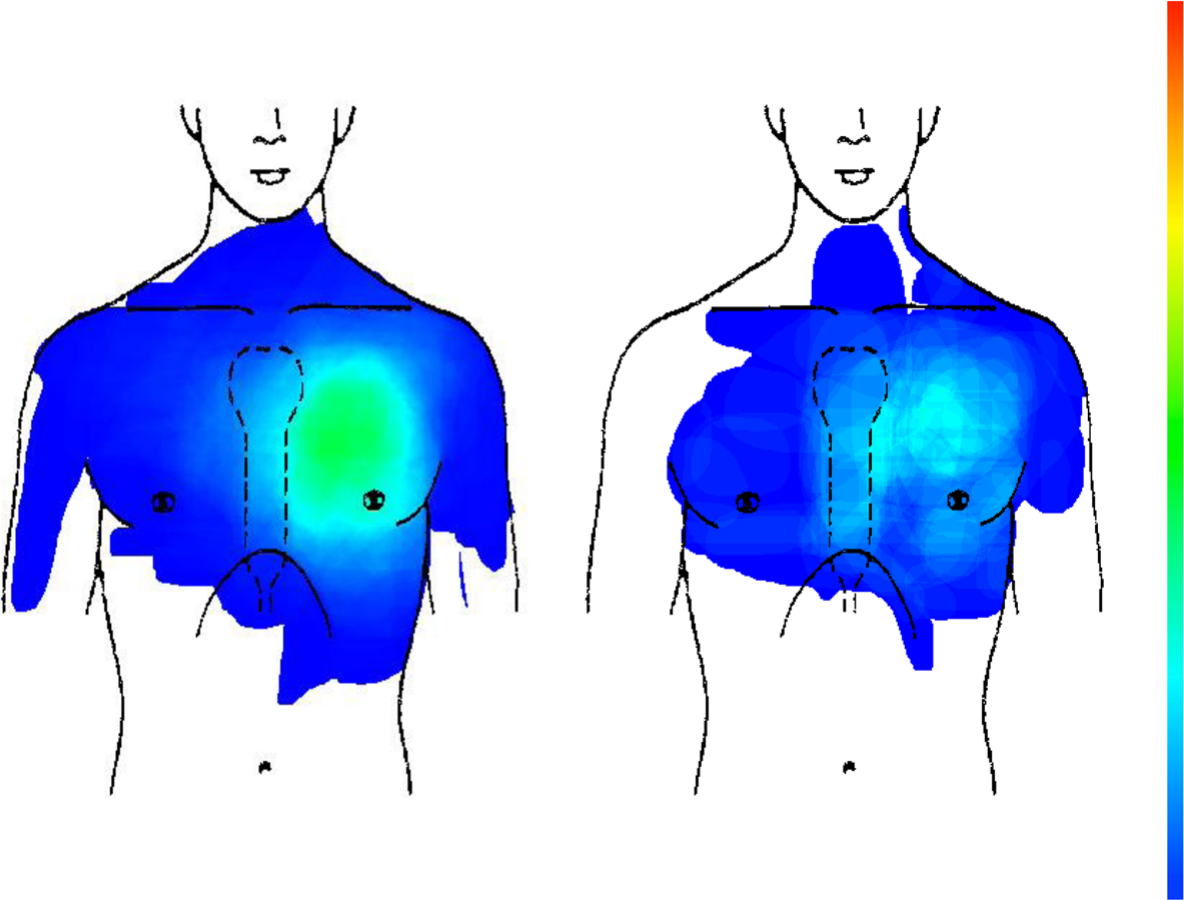
Patients with chest wall syndrome (CWS) who assume a cardiac origin of their pain (left picture, n = 298) compared with CWS patients who do not assume a cardiac origin (right picture, n = 199).

Pain localization in patients with CWS who assume a cardiac origin of their pain concentrates on the left anterior chest. Localization in CWS patients that do not assume a cardiac origin is more scattered, although also mainly situated on the left anterior chest. Distribution differs significantly (see Figure 7 and Table 2).

## Discussion

We examined in a large prospective primary care study whether pain localization is helpful to discriminate between CHD and other diseases in chest pain patients. Pain localizations of all major chest pain etiologies (CHD, CWS, GERD and psychogenic chest pain) were mainly situated on the left anterior chest and did not help to discriminate between CHD and other diseases.

Strengths of our study are a large primary care based consecutive sample which is highly representative, the prospective design and low drop-out rates during the follow up period. Study procedures such as random audits reduced the possibility of selection bias. An interdisciplinary team of PCPs and cardiologists provided a precise diagnosis as reference standard. As we did not cluster pain localization data but plotted the original drawings with the help of a specially designed computer program we could maintain highest data integrity for graphical and statistical analysis.

As we did not interfere with the work-up provided by participating PCPs, for some patients only limited clinical data were available to the reference panel. Since data from the original questionnaire, also including PCPs’ provisional diagnoses, were also used by the panel for decision making, there may be a degree of incorporation bias in regard to the final diagnoses [21].

We did not find pain localization helpful in discriminating between CHD and other diseases. This stands in contrast to Gencer et al. who analyzed a sample of 672 chest pain patients in primary care and found an association between substernal pain and CHD [22]. Several other studies could find no or only limited use of the localization of chest pain in predicting which patients would eventually have ACS or acute myocardial infarction (AMI) [9,23-27]. Cooke et al. examined chest pain characteristics in a highly selected patient population with chronic stable CHD and could also find no differences in pain localization [28]. Only one study in patients referred for coronary angiography found that pain to the left of the sternum occurs more frequently in patients with normal coronary arteries [29]. In a systematic review modeling the investigation of acute chest pain in primary care conducted by Mant et al., localization of chest pain was not helpful in ruling ACS in or out [11].

Chest wall syndrome (CWS) constitutes the most common etiology of chest pain in primary care [30,31]. The question whether pain localization is helpful to distinguish between CWS and CHD is therefore of high practical relevance for PCPs. From a pathophysiological point of view one would expect that pain localization of a higher number of patients with CWS would be more or less equally distributed with no preference for one side of the thorax.

However, our data show that CWS, like CHD, is mainly situated on the left anterior chest side, and that location does not discriminate between these two diseases. Our findings are supported by Verdon et al. who observed in a cohort of 300 primary care patients with CWS also the main pain localization on the left or median-left part of the chest wall [3]. Wise et al. describe in a selected population of 100 patients with negative coronary arteriography 69 patients with chest wall tenderness, most commonly situated in the sternal and left anterior chest wall area [32]. One has to assume that even in an 'unselected’ primary care population as observed in our study as well as by Verdon et al., there is already a certain selection effect. Due to public health campaigns the general population associates left thoracic pain mainly with the danger of CHD and will most likely contact a doctor more frequently than if the same pain occurred in any other thoracic area. This is supported by our sub-analysis presented in Figure 7 which compares CWS patients who assume a cardiac origin of their pain with CWS patients without this assumption. The first group shows a statistically significant more clustered pain distribution where the heart is situated.

Symptoms of GERD are a common complaint in primary care patients [33]. The pain caused by GERD can mimic the pain caused by CHD. In a study conducted by Davies et al. classical features of angina pectoris were equally common in CHD patients and patients with esophageal disease [34]. As both organs are situated near to each other and the resulting pain is in each case of visceral nature, one would expect few differences in pain localization, which is also supported by our data.

Psychogenic chest pain ranks among the 5 most frequent etiologies of chest pain in primary care [30,31]. Beside panic disorders, anxiety and depression prevail in these patients [35]. As these patients themselves often assume a cardiac origin they are in particular danger of receiving unnecessary further investigations. Pain location is classically described as uncharacteristic affecting multiple sites of the chest and being difficult to distinguish from CHD [36]. Our findings show nearly a complete overlap of pain regions in patients with psychogenic chest pain compared to CHD induced chest pain. On the one hand we would postulate similar self-selection mechanisms as already described above for CWS patients. Additionally it might be the very nature of psychogenic pain to be more projected towards the cardiac area. It could also be shown that myocardial infarction patients report left sided chest pain during their prodromal phase in the same frequency as a control group of patients with hyperventilation and/or functional complaints [37].

Finally we analyzed pain distribution in male and female CHD patients and could also find no difference. There are many studies that have examined gender differences in symptom presentation, mainly for ACS or AMI [38-40]. All of these have been performed in emergency departments and the authors did not specifically investigate left sided chest pain, but other pain regions. Major differences in pain distribution were not described.

In summary our results show that chest pain localization is neither helpful in discriminating CHD patients from other patients nor is it helpful to identify or exclude other chest pain etiologies. While in some diseases there might be an a priori high overlap of pain localization (e.g. CHD and GERD), in other conditions (like CWS or psychogenic chest pain) pain localization might trigger consultation of a health care provider. Consequently, the diagnostic value of pain localization in this instance is already 'used up’ and no longer of diagnostic value in the primary care setting. Similar phenomena of self-selection bias have been described for other clinical settings [41,42] and may have contributed to our findings.

## Conclusions

In contrast to the information still provided in many medical textbooks pain localization is not helpful in discriminating CHD from other common chest pain etiologies. Doctors should focus more on other clinical characteristics when evaluating chest pain patients [43].

## Competing interests

The authors declare that they have no competing interests.

## Authors’ contributions

NDB formulated the research question, designed the study and supervised its conduct. NDB, JH, KB, PS, EH and SB were involved in analysis and interpretation of data. SB drafted the article; NDB, JH, KB, PS and EH revised it critically. SB had full access to all of the data in the study and takes responsibility for the integrity of the data and the accuracy of the data analysis. All authors approved the final manuscript.

## Pre-publication history

The pre-publication history for this paper can be accessed here:

http://www.biomedcentral.com/1471-2296/14/154/prepub
